# Supplementary material for: A Multicenter, Investigator-Blinded, Randomized Controlled Trial to Assess the Efficacy of Calf Neuromuscular Electrical Stimulation Program on Walking Performance in Peripheral Artery Disease: The ELECTRO-PAD Study Protocol
Source: J Clin Med. 2022 Dec 7;11(24):7261. doi: 10.3390/jcm11247261 (PMC9784261; doi:10.3390/jcm11247261)
Supplement: Supplementary file 1 [file jcm-11-07261-s001.zip › jcm-2036098-supplementary.pdf]

File S1. Spirit checklist for the ELECTRO-PAD study.

[Item 1 Spirit checklist] Descriptive title identifying the study design, population, interventions, and, if applicable, trial acronym

A multicenter, investigator-blinded, randomized controlled trial to assess the efficacy of calf neuromuscular electrical stimulation program on walking performance in peripheral artery disease: The ELECTRO-PAD study protocol

[Item 2b Spirit checklist] All items from the World Health Organization Trial Registration Data Set

Data category	Information
Primary registry<br>and trial identifying number	ClinicalTrials.gov<br>NCT03795103
Date of registration in primary registry	January 7, 2019
Secondary identifying numbers	35RC15_8961_ELECTROPAD<br>2016-A00971-50<br>PHRC N° API15R040
Source(s) of monetary or material support	French Ministry of Health
Primary sponsor	University of Rennes
Secondary sponsor(s)	None
Contact for public queries	Pr Guillaume Mahé, +33 (0)2 9928 9672,<br>guillaume.mahe@chu-rennes.fr<br>Dr Alexis Le Faucheur, +33 (0)2 9905 9419,<br>alexis.lefaucheur@ens-rennes.fr
Contact for scientific queries	Pr Guillaume Mahé, +33 (0)2 9928 9672,<br>guillaume.mahe@chu-rennes.fr<br>Dr Alexis Le Faucheur, +33 (0)2 9905 9419,<br>alexis.lefaucheur@ens-rennes.fr
Public title	Effect of a Neuromuscular Electrical Stimulation Program on Walking Capacity in Peripheral Artery Disease Patients (ELECTRO-PAD)
Scientific title	A multicenter, investigator-blinded, randomized controlled trial to assess the efficacy of calf neuromuscular electrical stimulation program on walking performance in peripheral artery disease
Countries of recruitment	France
Health condition(s) or problem(s) studied	Lower Extremity Peripheral Artery Disease
Intervention(s)	Active comparator: group of PAD participants following a 12-week program of neuromuscular electrical stimulation and receiving an information leaflet outlining tips for active living and walking.

	Placebo comparator: group of PAD participants only receiving an information leaflet outlining tips for active living and walking.
Key inclusion and exclusion criteria	***Inclusion Criteria:***<br>PAD participants<br>• Age > 40 years old;<br>• Subjects with Lower Extremity Peripheral Artery disease (LEPAD). LEPAD is defined by the presence of at least one of the following criteria:<br>✓ History of revascularization in the lower limbs due to LEPAD; OR<br>✓ Ankle brachial index (ABI) of ≤ 0.90[1]; OR<br>✓ ABI or ankle systolic blood pressure decrease during recovery from treadmill walking test > 20 % or > 30 mmHg, respectively; OR<br>✓ Toe-brachial index ≤0.70 if ABI cannot be measured and if incompressible arteries are suspected;<br>• Complain of exertional calf pain (fatigue, discomfort or cramping) that can begin or not at rest, causes the participant to stop walking and relieves or lessens within 10 minutes of rest (assessed using the San Diego questionnaire AND confirmed during treadmill testing);<br>• Pain (fatigue, discomfort or cramping) is mainly located at the calves' level;<br>• Maximal walking distance on treadmill < 300 m (treadmill protocol 3.2 km/h, 10% grade);<br>• Subject receiving from at least one month the recommended medical therapy for LEPAD management (antiplatelet therapy and statin medication);<br>• Obtained informed consent.<br><br>Healthy volunteers [ancillary study #2]<br>• Age > 50 years old;<br>• Absence of pain reported in the lower limbs during walking as supported by a negative score on the San Diego Questionnaire;<br>• Absence of any functional limitation during the treadmill walking test: 15 min of walking at 3.2 km/h and 10% slope;<br>• ABI ≥1.00 and ≤1.40;<br>• Obtained informed consent.<br><br>***Exclusion Criteria:***<br>PAD participants<br>• Patients with a pacemaker or defibrillator;<br>• Patients with acute or critical limb ischemia;<br>• Ambulation limited by exertional symptoms other than intermittent claudication (e.g., dyspnea or angina pectoris);<br>• Ambulation limited by exertional symptoms indicative of intermittent claudication but affecting muscles in the lower extremities other than the calves;

- Contraindication to exercise testing according to the American Heart Association and the American College of Sports Medicine;
- Major cardiovascular event (myocardial infarction or stroke) or major surgery within the previous three months before inclusion;
- Female patients who are pregnant, planning to become pregnant, or lactating;
- Known presence of an aneurysm of the abdominal aorta > 4cm or an aneurysm of the iliac artery >1.5cm;
- Patient subject to legal protection (guardianship or tutelage measure) and persons deprived from their liberty (according to French law);
- Simultaneous participation to another ongoing clinical research protocol;
- Unwilling or unable to engage in the completion of a 12-week program;
- Any planned event(s) that could interfere with the completion of the protocol: e.g., extended holidays preventing the completion of the intervention or planned hospitalization for a prolonged period;
- Body mass > 160kg (may exceed treadmill limit);
- Inability to understand and sign informed consent forms due to cognitive or language barriers;
- LEPAD due to other causes than atherosclerosis.

Healthy volunteers [ancillary study #2]

- Contraindication to walking;
- ABI <1.00 or >1.40;
- Inability to obtain a measure of ABI due to uncompressible arteries.
- Limitation(s) and/or symptoms during the treadmill walking test;
- Treadmill walking test uncompleted;
- Presence of hypertension, heart failure, angina pectoris, diabetes, chronic obstructive pulmonary disease, supported by the presence of a medical treatment and the medical history;
- Presence of conditions likely to cause a functional limitation in walking and/or significant modification of physiological responses to the exercise: current or former smoker from less than 6 months, cancer (ongoing), Parkinson's disease, renal failure (ongoing), supported by the presence of a medical treatment and the medical history;
- History of cardiovascular disease (heart failure, stroke, myocardial infarction) reported by the patient;
- Female volunteers who are pregnant, planning to become pregnant, or lactating;
- Volunteers subject to legal protection (guardianship or tutelage measure) and persons deprived from their liberty (according to French law);

	• Simultaneous participation to another ongoing clinical research protocol.
Study type	Interventional<br>Allocation: randomized<br>Intervention model: parallel assignment<br>Masking: single blind (investigator, outcomes assessor)<br>Primary purpose: treatment
Date of first enrolment	September 2019 for PAD participant<br>January 2019 for healthy participant (ancillary study)
Target sample size	80 PAD participants and 40 healthy participants (ancillary study)
Recruitment status	Last participant recruited. Data collection on-going
Primary outcome(s)	Change in treadmill walking distance to maximal leg pain [Time Frame: 12 weeks]<br>• Comparison of change in treadmill maximal walking distance (i.e., to maximal leg pain) after 12 weeks between NMES group and control group (between visit #1 and visit #2)
Key secondary outcomes	1. Change in treadmill walking distance to onset of leg pain [Time Frame: 12 weeks]<br>• Comparison of change in treadmill pain-free walking distance (i.e., to onset of leg pain) between visit #1 and visit #2, and between NMES and control group<br><br>2. Change in 6-minute total walk distance [Time Frame: 12 weeks]<br>• Comparison of change in 6-minute total walking distance between visit #1 and visit #2, and between NMES and control group<br><br>3. Change in outdoor walking capacity [Time Frame: 12 weeks]<br>• Comparison of change in outdoor walking capacity between visit #1 and visit #2, and between NMES and control group<br><br>4. Change in the Walking Impairment Questionnaire score [Time Frame: 12 weeks]<br>• Comparison of change in the Walking Impairment Questionnaire (WIQ) score between visit #1 and visit #2 and between NMES and control group<br><br>5. Change in the Short Form General Health Survey (SF-36) scores [Time Frame: 12 weeks]<br>• Comparison of change in the Short Form General Health Survey (SF-36) scores between visit #1 and visit #2, and between NMES and control group<br><br>6. Change in the Peripheral Artery Questionnaire (PAQ) scores [Time Frame: 12 weeks]

	• Comparison of change in the Peripheral Artery Questionnaire score between visit #1 and visit #2, and between NMES and control group<br><br>7. Change in daily physical activity level assessed by accelerometry [Time Frame: 12 weeks]<br>• Comparison of change in daily physical activity level assessed by accelerometry between visit #1 and visit #2 and between NMES, and control group<br><br>8. Change in ankle brachial index [Time Frame: 12 weeks]<br>• Comparison of change in ankle brachial index between visit #1 and visit #2, and between NMES and control group<br><br>9. Change in delta from resting oxygen pressure (DROP) using $TcPO_2$ during treadmill walking test [Time Frame: 12 weeks]<br>• Comparison of change in $TcPO_2$ DROP during treadmill walking test between visit #1 and visit #2 and between NMES and control group<br><br>10. Number of patients submitted to a revascularization procedure [Time Frame: 12 weeks]<br>• Comparison in the number of patients submitted to a revascularization procedure between visit #1 and visit #2, and between NMES and control group<br><br>11. Change in results of contrast imaging with laser granularity (skin microvascular function) [Time Frame: 12 weeks]<br>• Comparison of change in skin blood flow variation from post-occlusive hyperemia and local thermal hyperemia tests using laser speckle contrast imaging, only for PAD patients included in Rennes [ancillary study #1], between visit #1 and visit #2 and between NMES and control group<br><br>12. Change in maximum walking distance according to the location of the arterial obstruction [Time Frame: 12 weeks]<br>• Change in maximum walking distance according to the location of the arterial obstruction using scan images between visit 1 and visit 2 and between NMES and control group<br><br>13. Percentage of physiological response achieved by patients [Time Frame: 12 weeks]<br>• Percentage of physiological response achieved by patients for all outcome measures (comparison with parameters of volunteers without any cardiac or vascular disease) between all visits

[1]: PAD is defined by an ankle–brachial index (ABI; the ratio of the systolic blood pressure at the ankle to the systolic blood pressure in the arm) of 0.90 or less. An ABI is considered as "borderline" between 0.91 and 0.99, "normal" between 1.00 and 1.40, or "noncompressible" when >1.40 (see reference #3 in the manuscript, Gerhard-Herman et al., 2017).

[Item 3 Spirit checklist] Date and version identifier

Issue Date: March 22, 2022
Protocol Amendment Number: 10.0
The list of changes made relative to the previous protocol version can be found at: https://www.clinicaltrials.gov/ct2/history/NCT03795103

[Item 4 Spirit checklist] Sources and types of financial, material, and other support

See text "Fundings".

[Item 5a Spirit checklist] Names, affiliations, and roles of protocol contributors

See text "Authors contributions".

[Item 5b Spirit checklist] Name and contact information for the trial sponsor

Trial Sponsor: University Hospital of Rennes
Sponsor's Reference:
Contact name: Nicolas MEVEL
Address: CHU de Rennes - Hôpital de Pontchaillou. 2, rue Henri le Guilloux. 35033 Rennes cedex 9 – France.
Telephone: +33 2 99 28 25 55
Email: dri@chu-rennes.fr

[Item 5c Spirit checklist] Role of study sponsor and funders, if any, in study design; collection, management, analysis, and interpretation of data; writing of the report; and the decision to submit the report for publication, including whether they will have ultimate authority over any of these activities

See text "Fundings".

[Item 5d Spirit checklist] Committees

- **Scientific committee**
  - ✓ Roles: ensure the study follow-up, final decision for discontinuing or modifying allocated interventions for a given trial participant if one or more predefined criteria are met (see item 11b), to discuss and propose plan for results presentation.
  - ✓ Members: Pr Guillaume Mahé (principal investigator), Dr Alexis Le Faucheur (associated scientist), M. Pierre Jéhannin who was replaced (contract end) by M. Adrien Chanteau (research associate: data collection, participants follow-up).
- **Lead Investigators**

- Roles: responsible for identification, recruitment, data collection and completion of e-CRFs in each participating center.
- Members : Dr Clément Hoffmann, Pr Alessandra Bura-Rivière, Pr Samir Henni, Dr Damien Lanéelle, and Pr Guillaume Mahé.

- **Steering committee**
  - Roles: Agreement of final protocol, recruitment of patients and liasing with principal investigator, reviewing progress of study and if necessary, agreeing changes to the protocol.
  - Members: Scientific committee members, Dr Marie-Laure Gervais, Pr Bruno Laviolle.
- **Trial Management Committee**
  - Roles: Study planning, organization of steering committee and contacts with steering committee, budget administration and contractual issues with individual centers, advice for lead investigators, audit of 6 monthly feedback forms and decide when site visit to occur.
  - Members: Scientific committee members, Dr Marie-Laure Gervais, Pr Bruno Laviolle.
- **Data Manager**
  - Roles: Maintenance of trial information technology and data entry (eCRF), data verification, randomization.
  - Members: Ms Garance Lagadic
- **Methodologist**
  - Roles: ensure methodological reliability of study design.
  - Members: Pr Bruno Laviolle
- **Biostatistics committee**
  - Roles: ensure reliability of biostatistics analysis plan and reliability of biostatistics methods used.
  - Members : Pr Guillaume Mahé, Dr Alexis Le Faucheur, Bruno Laviolle, Pauline Blanc-Petitjean.

[Item 6a Spirit checklist] Description of research question and justification for undertaking the trial, including summary of relevant studies (published and unpublished) examining benefits and harms for each intervention

See text “Introduction” section, and see the following reference:

Jéhannin, P., Craughwell, M., Omarjee, L., Donnelly, A., Jaquinandi, V., Mahé, G., & Le Faucheur, A. (2020). A systematic review of lower extremity electrical stimulation for treatment of walking impairment in peripheral artery disease. Vascular medicine (London, England), 25(4), 354–363. https://doi.org/10.1177/1358863X20902272

[Item 6b Spirit checklist] Explanation for choice of comparators

See text “Discussion” section, and see the following reference:

Jéhannin, P., Craughwell, M., Omarjee, L., Donnelly, A., Jaquinandi, V., Mahé, G., & Le Faucheur, A. (2020). A systematic review of lower extremity electrical stimulation for treatment of walking impairment in peripheral artery disease. Vascular medicine (London, England), 25(4), 354–363. https://doi.org/10.1177/1358863X20902272

[Item 7 Spirit checklist] Specific objectives or hypotheses

The primary objective is listed in the manuscript. Below are presented the main secondary objectives of the ELECTRO-PAD study.

Key Secondary Objectives

For PAD participants, key secondary objectives are to determine the effect of NMES over a three-month home-based program on the change of:

a) the total walking distance achieved following the 6-minute walk test.
b) the walking ability in natural walking conditions assessed by Global Positioning System (GPS) measurement.
c) the walking ability perceived by the patient assessed by questionnaire (WIQ).
d) the quality of life assessed by questionnaire (SF-36 and PAQ).
e) the level of spontaneous physical activity assessed objectively by wearable activity monitors.
f) the degree of ischemia assessed by exercise oximetry during treadmill walking test (only for equipped centers).
g) the ankle-brachial index.
h) endothelial function assessed by granularity contrast imaging by laser method (Laser Speckle), only for PAD participants included in Rennes due to material availability [ancillary study #1].
i) the maximum walking distance depending on the location of the arterial obstruction as determined by a scan, and only in PAD participants that benefit from this medical exam as a part of their disease management.

Other Secondary Objectives

For healthy participants (ancillary study #2), a secondary objective is to determine the normal range for most of the different outcome measures that are performed in PAD participants.

[Item 8 Spirit checklist] Description of trial design including type of trial (e.g., parallel group, crossover, factorial, single group), allocation ratio, and framework (e.g., superiority, equivalence, non-inferiority, exploratory)

See text “Methods and analysis / Study Design”.

[Item 9 Spirit checklist] Description of study settings (e.g., community clinic, academic hospital) and list of countries where data will be collected. Reference to where list of study sites can be obtained

See text “Methods and analysis / Recruitment, randomization, and blinding”.

[Item 10 Spirit checklist] Inclusion and exclusion criteria for participants. If applicable, eligibility criteria for study centres and individuals who will perform the interventions (e.g., surgeons, psychotherapists)

Inclusion and exclusion criteria for participants are provided above in Item 2b.

[Item 11a Spirit checklist] Interventions for each group with sufficient detail to allow replication, including how and when they will be administered

See text “Methods and analysis / NMES Intervention”.

[Item 11b Spirit checklist] Criteria for discontinuing or modifying allocated interventions for a given trial participant (e.g., drug dose change in response to harms, participant request, or improving/worsening disease)

PAD participants

The occurrence of the criteria described below may lead to the interruption or total discontinuation of the program for the NMES group. However, patient follow-up will be continued to allow the collection and intention-to-treat analysis of the primary endpoint.

- Patients requiring pacemaker or defibrillator within the first three months.
- For the primary endpoint, patients who underwent revascularization (surgical or endovascular) during the first three months.
- Any health events during the first three months that no longer enable the patient to follow the protocol.
- Skin burns reported during electrostimulation sessions.
- Skin allergy due to the electrostimulation electrodes.

Healthy participants [ancillary study #2]

- Any health issue occurring during the inclusion period and preventing the participant to complete the protocol.

[Item 11c Spirit checklist] Strategies to improve adherence to intervention protocols, and any procedures for monitoring adherence (e.g., drug tablet return; laboratory tests)

See text “Methods and analysis / NMES Intervention”.

[Item 11d Spirit checklist] Relevant concomitant care and interventions that are permitted or prohibited during the trial

Authorized treatments

PAD participants will be asked to systematically call the principal investigator center to inform of any change in drug treatment. Furthermore, as part of the follow-up of the groups via phone calls, the research associate will also ask patients about this. The case where applicable, the information will be noted directly on the e-CRF. Where possible, it is recommended that the drug treatment is not modified during the protocol. However, if necessary medical treatment, all drug treatments are potentially authorized during the protocol (see below). The same will apply to healthy voluntary participants in the event of medical necessity.

Unauthorized processing

Regarding participants with PAD, as indicated above, in case of medical event, there is no treatment drugs not authorized during the protocol. The prescription of certain drugs can potentially induce a change in the maximum walking distance if they occur during protocol: ACE inhibitor, vasodilator, statin, and platelet aggregation inhibitor. If these drugs are prescribed, the principal investigator will contact the primary care physician to judge the need for the immediacy of the treatment. Patient follow-up will be continued to allow the collection and intention-to-treat analysis of the primary outcome. A modification of the prescription will be analyzed as a potential confounding factor in the statistical analyzes (per-protocol analysis). For patients in the NMES group, the need to implant a pacemaker or defibrillator will cause stopping the program if this takes place during the interventional phase of the study, i.e., during the first three months when carrying out the NMES program. Patient follow-up will be pursued to allow the collection and intention-to-treat analysis of the primary outcome.

Emergency treatment
Participation in the protocol does not contraindicate the initiation of emergency treatment(s) or procedure(s). If any emergency treatment or procedure occur, the principal investigator will quickly request the scientific committee. Together, they will judge whether this treatment emergency is likely to interrupt the implementation of the current program temporarily or totally for patients allocated to the NMES arm. If the emergency treatment is likely to induce a significant change in the patient's maximum walking distance, taking this treatment will be analyzed as a potential confounding factor in the statistical analyzes (per protocol analysis). Patient follow-up will be pursued to allow the collection and intention-to-treat analysis of the primary outcome.

[Item 12 Spirit checklist] Primary, secondary, and other outcomes, including the specific measurement variable (e.g., systolic blood pressure), analysis metric (e.g., change from baseline, final value, time to event), method of aggregation (e.g., median, proportion), and time point for each outcome. Explanation of the clinical relevance of chosen efficacy and harm outcomes is strongly recommended

See text “Methods and analysis / Primary and secondary outcomes”, and above Item 2b Spirit.

[Item 13 Spirit checklist] Time schedule of enrolment, interventions (including any run-ins and washouts), assessments, and visits for participants

See text “Methods and analysis / Recruitment, randomization, and blinding”, and Figure 1.

[Item 14 Spirit checklist] Estimated number of participants needed to achieve study objectives and how it was determined, including clinical and statistical assumptions supporting any sample size calculations

See text “Methods and analysis / Sample size”.

[Item 15 Spirit checklist] Strategies for achieving adequate participant enrolment to reach target sample size

See text “Methods and analysis / Recruitment, randomization, and blinding”.

[Item 16a Spirit checklist] Method of generating the allocation sequence (e.g., computer-generated random numbers), and list of any factors for stratification. To reduce predictability of a random sequence, details of any planned restriction (e.g., blocking) should be provided in a separate document that is unavailable to those who enroll participants or assign interventions

- **Method of sequence generation ➔** Computerized random number generator.
- **Allocation ratio ➔** 1:1
- **Type of randomization ➔** Blocked randomization. The block size is not disclosed, to ensure concealment.

Note:
All randomization procedures were performed by the biometrics unit of the pharmacology department (INSERM, Clinical Investigation Centre 1414) of the coordinating center. This randomization was performed according to the number and future inclusion numbers. The different strata of randomization were as follows:

a) Stratified randomization by center and balanced 1:1 to determine the distribution of patients in each of the groups: CONT and ESM.
b) Stratified randomization by center and balanced 1:1 to determine the order of the treadmill test and the 6 min at each visit. The same order will be maintained for the same patient at visits 1 and 2.
This randomization was automatically performed on the Electronic case report form (e-CRF) and - as far as the allocation of patients to each group was concerned – was the distribution of patients in each group - it was only visible to the biometrics unit of the department of pharmacology department of Rennes, the clinical research associate (CRA) and the research associate working on the protocol.

[Item 16b Spirit checklist] Mechanism of implementing the allocation sequence (e.g., central telephone; sequentially numbered, opaque, sealed envelopes), describing any steps to conceal the sequence until interventions are assigned

Automatic randomization that was performed at the e-CRF level. The e-CRF is accessible online using EnnovClinical software, according to EU data protection regulation
The result of the randomization is only accessible to the data manager and the central engineer who manage the corresponding strategy kits for the patients. The patient will not know his or her assignment until after the inclusion visit, once receiving the postal package.

[Item 16c Spirit checklist] Who will generate the allocation sequence, who will enroll participants, and who will assign participants to interventions

See text "Methods and analysis / Recruitment, randomization, and blinding", and below.

- **Allocation sequence generation:** the biometrics unit of the pharmacology department (INSERM, Clinical Investigation Centre 1414) of the coordinating center.
- **Participants enrollment**: clinical investigator in each center.
- **Participants assignation to interventions:** research associate of the coordinating center.

[Item 17a Spirit checklist] Who will be blinded after assignment to interventions (e.g., trial participants, care providers, outcome assessors, data analysts), and how

See text "Methods and analysis / Recruitment, randomization, and blinding".

[Item 17b Spirit checklist] If blinded, circumstances under which unblinding is permissible, and procedure for revealing a participant's allocated intervention during the trial

Unblinding is deemed to be necessary in case of a PAD participant in the NMES group requiring pacemaker or defibrillator during the program. The presence of this criteria will lead to NMES program discontinuation for the participant [Item 11b].

[Item 18a Spirit checklist] Plans for assessment and collection of outcome, baseline, and other trial data, including any related processes to promote data quality (e.g., duplicate measurements, training of assessors) and a description of study instruments (e.g., questionnaires, laboratory tests) along with their reliability and validity, if known. Reference to where data collection forms can be found, if not in the protocol

See text, "Methods and Analysis section / Outcomes assessment (visits #1 and #2)".

[Item 18b Spirit checklist] Plans to promote participant retention and complete follow-up, including list of any outcome data to be collected for participants who discontinue or deviate from intervention protocols

Both in the NMES and non-intervention control-group, phone calls are also conducted every two weeks (±4 days) notably for promoting participant retention and completing follow-up.

[Item 19 Spirit checklist] Plans for data entry, coding, security, and storage, including any related processes to promote data quality (e.g., double data entry; range checks for data values). Reference to where details of data management procedures can be found, if not in the protocol

Data forms and data entry
All data will be entered electronically on e-CRF (EnnovClinical software). This will be done at the participating site where the data originated. A sponsor CRA will have access on e-CRF and on data of medical file on site with specific access, later for quality control (monitoring). After data monitoring, data can't be modified without sponsor agreement. Electronic queries will be edited to correct any data.

Data Transmission and Editing
The construction and management of the e-CRF are ensured by the CIC Inserm 1414 which is ISO 9001 certified. The CIC Inserm 1414 uses the Ennov Clinical® solution from Ennov which is also ISO 9001 certified.

The data entry screens will resemble the paper forms approved by the sponsor and the scientific committee. Data integrity will be enforced through a variety of mechanisms. Referential data rules, valid values, range checks, and consistency checks against data already stored in the database (i.e., longitudinal checks) will be supported. The option to choose [sic] a value from a list of valid codes and a description of what each code means will be available where applicable. Checks will be applied at the time of data entry into a specific field and/or after the data monitoring. Modifications to data written to the database will be documented through either the data change system or an inquiry system. Data entered into the database will be retrievable for viewing through the data entry applications. The type of activity that an individual user may undertake is regulated by the privileges associated with his/her user identification code and password.

All information about the database including changes and the list of consistency checks are listed in the data validation plan.

Data Discrepancy Inquiries and Reports
Additional errors will be detected by programs designed to detect missing data or specific errors in the data. These errors will be summarized along with detailed descriptions for each specific problem in Data Query Reports, which will be sent to the participating site. The investigator who receives the inquiry will respond by checking original sources to determine the correction, modifying the original data form entering a response to the query. Note that it will be necessary for the investigator to respond to each inquiry received to obtain closure on the queried item. Written documentation of changes will be available via electronic logs and audit trails.

At the end of the study, a report summarizing the quality of the data is written by the data manager. This report contains all deviations from the protocol, missing data, and any residual inconsistencies. This document will be used as a basis for the statistical analysis plan.

Security and Back-Up of Data

The database is hosted on a secure server managed by Ennov via a specialized partner, OVH. OVH is certified ISO 9001 and ISO 27001.

All security and backup procedures are listed in a PIA specific to the use of Ennov Clinical® written by the CIC Inserm 1414.

The main elements of the PIA are:

- There are two ways to access the database (1- via CS Online for the participating sites, only the persons mandated by the data controller will have exclusive access to the eCRF, with a personal identifier and a password; 2- via Remote Desktop Gateway which allows authorized external users to access the Ennov data center network from an internet connection. The combination of RDP and HTTPS protocols provides a secure and encrypted connection. Only persons authorized by the data controller will have exclusive access to the eCRF via the RDP access to perform quality control, monitoring, and follow-up of the study.
- Measures are in place to ensure data confidentiality during the development of the computer application (1. The development and production environments are distinct; 2. The personnel assigned to development and management/operations tasks are distinct; 3. Software development is carried out on fictitious data).
- Storage media intended for destruction are subject to a special protection procedure by the hosting company OVH, when the disks are no longer used.
- Entitlement profiles define the functions or types of information accessible to a user by a logical access control by password configured and defined in the CSAdministrator module and by an application administrator within the institution, by means of "client" software certificates.
- The application implements processes:
  * Encryption of stored personal data = Algorithm (e.g. 3DES): AES Key length = 256bits.
  * Securing the transport of personal data = Security protocol: SSL.
  * Authentication of the recipient or "server" = Method and trade name: THAWTE SSL Certificate and domain name.
  * Sender or "client" authentication = Method and trade name: electronic signature and connection identifier
- The outsourced servers hosting the data and its software are under Windows server 2012 R2 DATACENTER operating system and the database system implemented is ORACLE. The servers are protected by 1- anti-virus software installed on all workstations involved in processing; 2- network compartmentalization with filtering rules (DMZ, firewall); 3- Internet exchanges (Web including portal, file transfer, email, etc.) using cryptographic protocols and mechanisms = HTTPS protocol, SSL; 4- firewalls).
- Backups are made by Ennov (1- Type of media: DISK; 2- Frequency: daily with 28 days retention; 3-Physical security of media location: DATACENTER; 4- Cryptographic mechanisms (storage and/or transport) used: Veeam solution; 5- Backup is managed by Ennov Clinical®; data is duplicated on two separate sites from the production environment).
- Server maintenance is performed by Ennov with measures to ensure data confidentiality during software or equipment maintenance operations (1- Maintenance operations are recorded in a handheld computer system ; 2- Software or equipment are remotely maintained ; 3- Security measures applied during these operations: secure connection via RDS gateway ; 4- Special procedure if remote maintenance requires access to personal data files: confidentiality commitment by IT administrators ; 5- Access by Ennov staff to the data is controlled by Ennov Access by Ennov staff is done through a nominative account).

The data will be kept in an active database, then archived for the period of time stipulated by the legislative and regulatory provisions in force concerning data from research involving the human person.

[Item 20a Spirit checklist] Statistical methods for analyzing primary and secondary outcomes. Reference to where other details of the statistical analysis plan can be found, if not in the protocol

See text "Methods and analysis / Data collection, management, and analysis", and summary table below.

Variable/Outcome	Hypothesis	Outcome Measure	Method of analysis
**Primary outcome**			
Change in treadmill maximal walking distance (MWD) at 3 months.	Increase of MWD in NMES group	Treadmill MWD [continuous]	Two sample, 2-sided t tests (or Mann–Whitney U test)
**Secondary outcomes**			
Change in treadmill pain-free walking distance (PFWD)	Increase of treadmill PFWD in NMES group	Treadmill PFWD [continuous]	Two sample, 2-sided t tests (or Mann–Whitney U test)
Change in 6-minute total walk distance	Increase of 6-minute total walk distance in NMES group	6-minute total walk distance [continuous]	Two sample, 2-sided t tests (or Mann–Whitney U test)
Change in maximal outdoor walking capacity	Increase of maximal outdoor walking MET·min[a] in NMES group	Maximal walking MET·min [continuous]	Two sample, 2-sided t tests (or Mann–Whitney U test)
Change in the Walking Impairment Questionnaire (WIQ) total score or sub-scores	Increase of the WIQ total score or sub-scores in NMES group	The WIQ total score and sub-scores [discrete]	Two sample, 2-sided t tests (or Mann–Whitney U test)
Change in the Short Form General Health Survey (SF-36) scores	Increase of the SF-36 scores in NMEs group	Short Form General Health Survey (SF36) scores [discrete]	Two sample, 2-sided t tests (or Mann–Whitney U test)
Change in the Peripheral Artery Questionnaire (PAQ) scores	Increase of the PAQ scores in NMES group	The Peripheral Artery Questionnaire (PAQ) scores [discrete]	Two sample, 2-sided t tests (or Mann–Whitney U test)
Change in daily physical activity level assessed from accelerometry[b]	Increase of daily physical activity level in NMES group	Daily physical activity level [continuous]	Two sample, 2-sided t tests (or Mann–Whitney U test)
Change in ankle brachial index (ABI)	Increase of ABI in NMES group	ABI [continuous]	Two sample, 2-sided t tests (or Mann–Whitney U test)

Change in $TcPO_2$ DROP during treadmill walking test	Increase of $TcPO_2$ DROP in NMES group	$TcPO_2$ DROP [continuous]	Two sample, 2-sided t tests (or Mann–Whitney U test)
Proportion of patients submitted to a revascularization procedure	Fewer proportion of patients submitted to a revascularization procedure in NMES group	Proportion of patients submitted to a revascularization procedure [dichotomous]	χ^2 test
Change in skin blood flow variation from post-occlusive hyperemia and local thermal hyperemia	Increase in skin blood flow variation (i.e., perfusion) in NMES group	Skin blood flow variation [continuous]	Two sample, 2-sided t tests (or Mann–Whitney U test)
Change in MWD according to the location of the arterial obstruction and the location of ischemic symptoms inducing walking limitation (i.e., unilateral, bilateral)	No hypothesis (descriptive only)	Treadmill MWD [continuous]	Two sample, 2-sided t tests (or Mann–Whitney U test) / Anova test (or Kruskal-Wallis test)[c]
Percentage of physiological response achieved by patients for all outcome measures, at both visits	No hypothesis (descriptive only)	See above, according to each outcome	See above, according to each outcome

[a] MET: Metabolic Equivalent of Task. See text for methods. The change for all other GPS parameters (continuous variables) will be also tested (see text, Methods section).
[b] Daily physical activity (PA) level mainly assessed by: mean time per day of total PA, mean time per day of light, moderate and vigorous PA, mean number of steps per day, mean number of steps per day (or mean daily stepping time) accumulated in different bouts duration, mean number of steps per day (or mean daily stepping time) accumulated in different cadence bands (See text, Methods section).
[c] Comparison with measures performed on healthy participants without any cardiac or vascular disease.
[d] According to the number of locations of the arterial obstruction.

[Item 20b Spirit checklist] Methods for any additional analyses (e.g., subgroup and adjusted analyses)

See text "Methods and analysis / Data collection, management, and analysis".

[Item 20c Spirit checklist] Definition of analysis population relating to protocol non-adherence (e.g., as randomized analysis), and any statistical methods to handle missing data (e.g., multiple imputation)

See text “Methods and analysis / Data collection, management, and analysis”.

[Item 21a Spirit checklist] Composition of data monitoring committee (DMC); summary of its role and reporting structure; statement of whether it is independent from the sponsor and competing interests; and reference to where further details about its charter can be found, if not in the protocol. Alternatively, an explanation of why a DMC is not needed

DMC was deemed not necessary considering that the present protocol is at low-risk and not complex in data collection.

[Item 21b Spirit checklist] Description of any interim analyses and stopping guidelines, including who will have access to these interim results and make the final decision to terminate the trial

No interim analyses are planned.

[Item 22 Spirit checklist] Plans for collecting, assessing, reporting, and managing solicited and spontaneously reported adverse events and other unintended effects of trial interventions or trial conduct

Not applicable, according to French legislation (RIPH2 study).

[Item 23 Spirit checklist] Frequency and procedures for auditing trial conduct, if any, and whether the process will be independent from investigators and the sponsor

Not applicable.

[Item 24 Spirit checklist] Plans for seeking research ethics committee/institutional review board (REC/IRB) approval

See text “Methods and analysis / Study Design”.

[Item 25 Spirit checklist] Plans for communicating important protocol modifications (e.g., changes to eligibility criteria, outcomes, analyses) to relevant parties (e.g., investigators, REC/IRBs, trial participants, trial registries, journals, regulators)

Any modifications to the protocol will require a formal amendment to the protocol. Such amendment will be agreed upon by the sponsor and approved by the French institutional ethical committee prior to implementation and notified to the health authorities in accordance with local regulations.
See also: [Item 3 Spirit checklist] Date and version identifier.

[Item 26a Spirit checklist] Who will obtain informed consent or assent from potential trial participants or authorized surrogates, and how (see Item 32)

See text “Methods and analysis / Recruitment, randomization, and blinding”.

[Item 26b Spirit checklist] Additional consent provisions for collection and use of participant data and biological specimens in ancillary studies, if applicable

See text “Methods and analysis / Study Design”.

[Item 27 Spirit checklist] How personal information about potential and enrolled participants will be collected, shared, and maintained in order to protect confidentiality before, during, and after the trial

Personal data of each patient will be collected in the medical file. Its access will be authorized only to investigational persons and to sponsor CRA for monitoring, only on site.
All data collected in the database are anonymized according to French Authority CNIL (French MR001 and General Data Protection Regulation (EU) 2016/679 benchmarks). Each patient will be anonymized using a code xx-yyy, where xx is the number of the investigation site and yyy the number of the patient in the site, associated with initials (NF scheme).

Logical access control

- Authentication to log in to personal Windows account (unique identifier, associated with a password): complex 12-character password with renewal every 3 months.
- Study data entry on a secure e-CRF – Ennovclinical
    - https Web page.
    - Individual access: allocation of a login and password that is regularly refreshed.
    - Separate access rights for the investigating centers (investigating physicians or TEC) and the sponsor.
    - Personal health data hosting.

Storage after the trial

Anonymized data on database will be sent to statisticians for analyze and sponsor for secure archiving for 15 years according to French law. Only the sponsor saves the database. Statisticians will destroy it after analyses.
In accordance with the provisions concerning the confidentiality of data to which the persons in charge of quality control of research involving the human person have access (article L.1121-3 of the French quality control of research involving the human person, in accordance with French public health code (article L.1121-3 of the Public Health Code), in accordance with the provisions concerning the confidentiality of information concerning the nature of the products, the tests, the persons who undergo them and the results obtained (article R.5121-13 of the Public Health Code), persons with direct access will take all necessary precautions to ensure the necessary to ensure the confidentiality of information relating to the products, the tests, the persons involved persons who take part in them and in particular with regard to their identity and the results obtained.
These persons, in the same way as the investigators themselves, are subject to professional secrecy (in accordance with the conditions defined by articles 226-13 of the conditions defined by articles 226-13 and 226-14 of the French Penal Code). During the research or at its conclusion, the data collected on the persons who are involved in the research and transmitted to the sponsor by the investigators (or any other specialist) will be made anonymous. Under no circumstances must the names of the persons concerned, or their addresses appear in clear text. Only the first letter of the subject's name and the first letter of his or her first name will be recorded, along with a coded number specific to the study indicating the center and the order of inclusion of the subjects in this center.

[Item 28 Spirit checklist] Financial and other competing interests for principal investigators for the overall trial and each study site

Name	Competing Interest
Alexis Le Faucheur	None
Pierre Jéhannin	None
Adrien Chanteau	None
Pauline Blanc-Petitjean	None
Alan Donnelly	None
Dr Clément Hoffmann	Bayer Healthcare (one-off communication); BMS/Pfizer (one-off communication)
Samir Henni	None
Prof Alessandra Bura-Rivière	Bayer Healthcare (consulting); BMS/Pfizer (consulting); Novartis (consulting)
Pr Adrien Kaladji	Medtronic (proctoring-consulting), Cook Medical (proctoring)
Damien Lanéelle	None
Pr. Guillaume Mahé	Bayer Healthcare (consulting); BMS/Pfizer (consulting); Novartis (consulting), LEO Pharma (consulting), Amarin (consulting); Amgen (consulting), Sanofi (consulting)

[Item 29 Spirit checklist] Statement of who will have access to the final trial dataset, and disclosure of contractual agreements that limit such access for investigators

Only sponsor and the Biostatistics committee will have access to final trial dataset.

[Item 30 Spirit checklist] Provisions, if any, for ancillary and post-trial care, and for compensation to those who suffer harm from trial participation

The Sponsor has taken out insurance covering, its own civil liability for the duration of the study as well as that of any physician involved in the study. It will also ensure full compensation for consequences of the research for the participant and his/her beneficiaries, unless he/she can prove that the damage was not caused by that the damage is not attributable to his or her fault or to that of

any other party involved, without the possibility of invoking the act of a third party or the voluntary withdrawal of the person who had initially agreed to take part in the research.

[Item 31a Spirit checklist] Plans for investigators and sponsor to communicate trial results to participants, healthcare professionals, the public, and other relevant groups (e.g., via publication, reporting in results databases, or other data sharing arrangements), including any publication restrictions

The results of the ELECTRO-PAD trial will be published in international peer-review journals. This includes a primary outcome paper and possible secondary outcome papers considering the extent of the results collected and the limitations imposed by scientific journals when submitting the primary outcome paper.

The results of the ELECTRO-PAD trail will be also presented as paper(s), abstract(s), oral or poster communication(s) in national and/or international conferences, if appropriate and timely. Some secondary outcome results could be also used for workshop (e.g., data from physical activity measurements with multiple metrics).

Each publication or communication will have to be approved by the Scientific Committee.

Each participant will receive an (oral or/and written) information presenting a summary of the results of the clinical trial.

[Item 31b Spirit checklist] Authorship eligibility guidelines and any intended use of professional writers

Authorship manuscript for the primary outcome paper (or any forms of communication) should rely on substantive contributions to the design, conduct, interpretation, and reporting of the ELECTRO-PAD clinical trial. Authorship criteria for manuscripts submitted for publication will be based on the International Committee of Medical Journal Editors.
Expected authors for the primary outcome paper are the members of the scientific committee, the statistician(s) and one clinical investigator from each participating center that will include participants. The clinical investigator from each participating center will be proposed by the Lead Investigators committee to the Scientific committee. In case of non-participation or very low rate of inclusion in a participating center, the Scientific committee retains authority to refuse authorship manuscript to a clinical investigator. Instead, the role in protocol design will be acknowledged in the published manuscript.

The same guidelines for authorship for secondary outcome manuscript or any forms of communication should apply. The main difference relies in that secondary publication could arise from additional and supplementary analyses that are not directly related to the primary aim of the clinical trial. In such cases, only individuals that make a substantive contribution to such additional analyses and results could be eligible for authorship. This may include other and new individual who add their skills to conduct such analyses. Again, the final approval for authorship will be given by the Scientific Committee.

The final version of the primary outcome manuscript will be submitted to an expert company for style and grammar corrections. Certification provided by the company will be available on simple request.

[Item 31c Spirit checklist] Plans, if any, for granting public access to the full protocol, participant-level dataset, and statistical code

Nothing more than what is presented in 31a.

[Item 32 Spirit checklist] Model consent form and other related documentation given to participants and authorized surrogates

- File S2: Information Letter and Consent Form given to the participants (French version).
- File S3: Handbook for the measurement of physical activity and outdoor walking capacity, both given to the NMES and control group (French version).
- File S4: Brochure with advice for engaging in a daily active lifestyle, both given to the NMES and control group (French version).
- File S5: Handbook for implementation and follow-up of the NMES program, given to the NMES group with unilateral calf symptoms (French version).
- File S6: Handbook for implementation and follow-up of the NMES program given to the NMES group with bilateral calf symptoms (French version).

[Item 33 Spirit checklist] Plans for collection, laboratory evaluation, and storage of biological specimens for genetic or molecular analysis in the current trial and for future use in ancillary studies, if applicable

Not applicable.
